# An Outlook on Physical and Virtual Sensors for a Socially Interactive Internet

**DOI:** 10.3390/s18082578

**Published:** 2018-08-06

**Authors:** Ngombo Armando, André Rodrigues, Vasco Pereira, Jorge Sá Silva, Fernando Boavida

**Affiliations:** 1 Centre for Informatics and Systems of the University of Coimbra (CISUC), University of Coimbra, 3030-290 Coimbra, Portugal; arod@dei.uc.pt (A.R.); vasco@dei.uc.pt (V.P.); sasilva@dei.uc.pt (J.S.S.); boavida@dei.uc.pt (F.B.); 2 Escola Superior Politécnica do Uíge, Universidade Kimpa Vita, Kondo Benze, Uíge, Angola; 3 Polytechnic Institute of Coimbra, ISCAC, 3040-316 Coimbra, Portugal

**Keywords:** Internet of Things, taxonomy, heterogeneity, social IoT

## Abstract

The Internet keeps changing at a rapid pace, driven mainly by the emerging concepts and applications that make it aware of the physical world and responsive to user context. The Internet of Things (IoT) concept is quickly giving way to more advanced and highly interactive environments that go well beyond the mere sensing of the physical world. Today, in addition to traditional electronic devices, IoT sensing/actuating includes both software and human-based entities. This paper provides an outlook on the future of sensing/actuating approaches on the Internet at large, which we see increasingly related to all kinds of socially interactive technologies. With these objectives in mind, we propose a taxonomy to deal with the heterogeneity of sensing/actuating approaches in IoT. We also analyse the state-of-the-art of Social Sensing. Finally, we identify open issues and associated research opportunities, the main ones being the integration of all sensing approaches, the combination of social sciences, engineering, and computing as enablers of context-aware, cognitive applications and, last but not least, the unified management of large sets of very heterogeneous sensors/actuators.

## 1. Introduction

IoT refers to the electronic paradigm where everything surrounding us—labelled “things”—have their corresponding virtual image in information technology systems and communicate with each other [1]. On the other hand, the IoT extends the computer’s network, conveying any sensed features of the physical world. IoT application covers daily activities for both monitoring and tracking purposes, including environmental monitoring, healthcare, critical infrastructure protection, automated diagnostics. Currently, there are more than 20 billion “things” in operation, and this figure will reach over 75 billion by 2025 [2]. Nevertheless, what is generically called the Internet of “Things” is already the networked connection of physical things and beyond. In fact, IoT includes people, processes, data and entities embedded with sensing/actuating capabilities to enable the representation of and to interact with entities [3]. Smart sensors, hereafter only referred to as sensors, are IoT’s founding blocks, and their core function is to gather data on target entities to enable their representation in an information system and, eventually, trigger actions using some kind of actuating device.

With reference to IoT’s communication model in Table 1 and the IEEE 1451 Standards Family [4,5], our study is focused on the sensing/actuating sublayer. Therefore, we tackle both conceptual and implementation approaches to describe the heterogeneity of entities in this sublayer. Also, by leveraging the potential of socialisation between entities as defined in [6], we propose a taxonomy for the heterogeneity of sensing/actuating entities. For the proposed taxonomy, we firstly organize the entities according to the target world where their activities are intended to be performed, i.e., into physical or virtual worlds. Secondly, we regroup entities according to their built-in nature, i.e., into electronic-based, human-based or software-based. We finally classify entities according to their interaction ability, i.e., standalone or social entities.

Considerable work has been done on the sensing/actuating IoT sublayer, either separately or exploring synergies between approaches. This paper surveys the concerned literature, with emphasis on the 2015–2017 period. Due to the amount and relevance of the work underway, it is essential to, on the one hand, survey existing initiatives and ongoing research and, on the other hand, identify the main challenges and research guidelines. These are the objectives of the current paper, whose main contributions are:

- a taxonomy for sensing/actuating entities in IoT, so as to establish a clear relationship between them;
- an analysis of the state-of-the-art on social sensing;
- identification of the main open issues in what concerns the use of sensing in a socially interactive Internet, as well as the resulting research opportunities.

The rest of the paper is organized as follows. In Section 2, we address the heterogeneity of sensing/actuation in an Internet made of things, computer resources, and people. In Section 3, we survey the Social Sensing paradigm, considering existing, relevant literature. Section 4 provides an outlook on the main challenges and open research directions in what concerns sensing/actuation for the coming years. The conclusions are presented in Section 5.

## 2. Dealing with Heterogeneity in IoT

In this section, we address the heterogeneity of sensing/actuating devices in an Internet made of things, computer resources, and people. We also present a taxonomy for IoT devices that derives from a thorough analysis of the existing literature.

### 2.1. Sensors and Actuators

**Figure 1:**
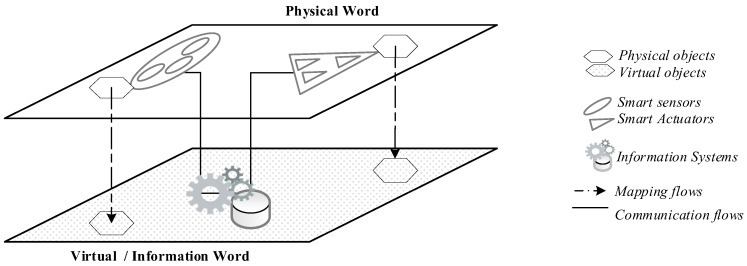
Technical overview of IoT enablers. Adapted from [6].

In what concerns the main entities in the sensing/actuating sublayer, we use the definitions in the OGC standards [8]. Thus, a sensor is an entity that retrieves the state of the sensed object and then pushes the collected data to one central processing and/or storage unit. An actuator is an entity that receives commands from a processing unit and executes an action on physical or virtual/information objects. International Telecommunication Union (ITU) technical overview of IoT enablers identifies networked entities—smart sensors/actuators—whose activities can be carried out in both physical and virtual worlds [7] (see Figure 1).

In this figure, virtual means in the realm of the pure information world. A web of physical and virtual sensing devices enables us to have a virtual representation of the physical world. For instance, consider the use of sensors to build a representation of air temperature variation in a given zone or the use of natural language processing techniques on written text in order to build a representation of someone’s traits of personality. In these scenarios, we may use weather electronics, software or humans as front-end “IoT devices” to interact with the target world. The activities in the physical world are mainly associated with traditional electronic devices in wireless sensor networks (WSN) [9]. These devices are purely based on microelectronic and mechanical systems to interact with the sensed object. However, humans are also able to objectively report some phenomena of the physical world, like how nice the weather is, the number of cars crossing a bridge, or how empty/full a bin really is in a smart city waste management system.

Data from electronic-based sensors can be used as is by the processing unit or can be fed into what is called virtual sensors. Indeed, contrary to both electronic and human entities, the activities in the virtual world are associated with software applications since they are capable of reporting events in the information world. Nitti et al. [10] surveyed the virtual objects in IoT from the perspective of the device, and even if the complexity of proposing a “standardized” one is still recognized, they give an interesting definition of Virtual Objects. To the authors, virtual objects are context-aware representations of the physical-electronic nodes in the real world, which augment the potentialities of the associated services and are strategic in the development of complex applications, addressing heterogeneity and scalability issues in IoT. There are, mainly, three types of representation models for virtual sensors. The first one is the Sensing-as-a-Service (SaaS) model [11,12,13], where both electronic nodes and their sensed data are put at the disposal of remote subscribers and operators. The second one is the estimation model [14,15]. Here, the virtual state of the environment is the result of a function that estimates its status based on a variety of third-party sensor inputs. The last virtual sensor model is the prediction model [16,17]. In this case, analytic techniques are used for predicting insights upon third-party sensors historical data.

Software programs cannot be used to fetch phenomena in humans’ minds directly. Therefore leveraging humans themselves arose as an exciting approach to fetch raw data from humans’ mind/virtual world. In this context, humans can be considered as sensors in their increasingly global societal environment, and are regarded as such in the literature [17,18]. Indeed, by analysing human activities in social media, for instance, it is possible to infer the emotions and mood of people while they consume sensed data and services [19]. To this end, tools such as sentiment analysis algorithms [20] and SVM (Support Vector Machine) techniques [21], enable us to give a numerical translation to the sensed data, that is, the text or any multimedia contents in a post.

Conversely, the IoT design in Figure 1 presents a web of actuating devices that can be used in both worlds. In fact, mechanical and electronic actuators can be looked at as the counterparts of electronic sensors, computational actuators can be looked at as the counterparts of software sensors, and human action can be looked at as the counterpart of human sensors. It is essential to consider the actuator concept in its broad sense, thus allowing for the adoption of a variety of ways for its implementation. In the traditional IoT context, an actuator may be implemented by mechanical action (e.g., open a tap or toggle an on/off switch) or even by human action in response to a notification. We can also see an actuator as a pure electronic signal via a LED state or a picture on a screen. Finally, we can consider an actuator as a computational operation to, for instance, fill a database or send notifications to change the value of variables in a remote computer program.

Given the concepts described in this subsection, in the next subsection, we will present our proposed taxonomy for Sensors and Actuators in IoT.

### 2.2. Taxonomy

The proposed sensors and actuators taxonomy is presented in Table 2, considering both the ITU technical overview for IoT in Figure 1 and the heterogeneity of devices described in the previous subsection.

Given the already mentioned heterogeneity in the IoT device layer, we start by classifying sensing/actuating devices according to the nature of the entities they are expected to interact with, namely, physical and virtual. As a consequence of considering both physical and virtual worlds, we then observe that sensing/actuating tasks can be performed by electronic-based, software-based, or human-based entities. In future, one may imagine human entities as a subset of a more extensive set. Indeed, we may label them as living-based nature, which will include any living source of information from the surrounding environment integrated into the IoT information system. Finally, we propose a classification of the IoT device entities according to the relationships they can establish among them. In fact, “things” connected to the Internet are different from the ‘things’ participating in the Internet of social networks [6]. Based on this observation, we can classify things as Standalone or Social. Standalone entities are those that only establish a direct upstream link to a node in the IoT system. As for social sensors, a relevant feature is that they are both data producers and data consumers. There are two broad classes of social sensors in the literature, which we labelled as Human social sensors and Non-human social sensors. Here, we use human operators to fetch the state of the environment and feed their observations into social networks.

**Figure 2:**
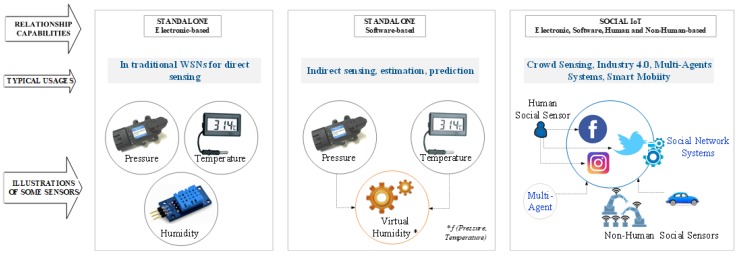
Some examples of sensor and actuator heterogeneity, and typical usage scenarios.

Figure 2 illustrates various types of sensor and actuator entities, including some typical usages. On the right, we can see a different type of social entities, in addition to human sensors. Considering the development of artificial intelligence (AI), we can have robots and computer programs emulating the humans’ role in such communities. For instance, in bot user systems (we have humans and agents interacting, cyber-physical systems cooperating in an Internet of Robotic Things (IoRT) [22], for Industry 4.0 purposes, or pure software-based agents for multi-agent system purposes [23] or high-frequency trading [24].

The snapshot in Table 3 provides some references along the various axes of our proposed taxonomy. Thus, the headers are the same as in Table 2, i.e., (i) the target activity object, which can be physical or virtual; (ii) the built-in nature of the entities, namely, hardware-based, software-based or human-based devices; and (iii) the relationship capabilities of the entities, namely, standalone or social devices. We can clearly see that research is increasingly considering sensing in its broader sense, i.e., looking well beyond the plain Internet of Things, into a socially interactive Internet.

In the next section, we will focus our attention on the social sensing paradigm as a way of exploring synergies between the sensing/actuating approaches described in our taxonomy.

## 3. The Social Sensing Paradigm

This section provides a focused yet comprehensive review of the Social Sensing paradigm. We start by addressing the definition of the social IoT concept and then proceed to identify and explain the main pieces of literature that deal with it.

### 3.1. Social IoT

Inspired by human activity in online social media, social sensors in IoT were proposed at the beginning of the 2010 decade and have since been gaining popularity [25,26,27,28]. The Social IoT concept (SIoT) is the result of applying the principles of IoT communication and networking to social media. Thus, social sensors are defined in literature considering the members of a community as both data producers and consumers. Hence, the Social Sensing paradigm is based on the increasing usage of social media to report the statuses of people and their environment. Saleem et al. [28] present a comprehensive state-of-the-art for SIoT and identify the main limitations of existing approaches. In an attempt to overcome current drawbacks, the authors propose the concept of recommendation services among various IoT applications. However, in their sample application scenario, humans are not considered to be both SIoT beneficiaries and enablers at the designed Perception Layer. We reiterate that what is generically called IoT is already a networked environment of things and beyond, since it includes people, processes, data, and things [3]. Thus, nowadays, we believe that the social sensing paradigm must involve both non-human entities and humans, as members of a social community. In fact, human social sensors are members of a community that shares contextualised data via a dedicated application, a web application, or an online social network. Software-based entities are to be included in SIoT since they can have social behaviour, as we saw in Section 2.

In SIoT, the trustful entities community is established according to five criteria:

- The parental relationship, in which the homogeneous entities originated in the same period by the same manufacturer can be considered as part of the same community;
- The co-location relationship, in which the entities of a delimited environment can be considered as part of the same community;
- The co-work relationship, in which the entities that collaborate in the same IoT application can be considered as part of the same community;
- The ownership relationship, in which the entities belonging to the same user can be considered as part of the same community;
- The social relationship, in which the entities belonging to owners that have a social affinity, e.g., friends, can be considered as part of the same community.

### 3.2. The Social Sensing Paradigm in the Literature

Bachiller et al. [19] propose a middleware solution to model and represent users in social sensing applications. The approach is based on two elements called User Component and User Bindings. The former models the user as a software component, allowing developers to deal with mobility between contexts. The latter enables the middleware to support multiple communication channels, including the association of users with sensing applications, irrespectively of the user network and/or device. According to the authors, humans may contribute to values (as sensors) and actions (as actuators), while also consuming sensing services. Thus, the integration of people into sensing systems has the potential to increase scale and reduce costs. Since an increasing number of people simultaneously use multiple communication devices (e.g., computer, smartphones, tablet), they argue that using online social networks as higher-level communication channels significantly improves the availability of the users in a participatory sensing scenario. In their study, participatory sensing is defined as a way of including users in distributed applications, enabling data to be acquired from both users and mobile phones’ sensors.

An IoRT-based neural network control scheme is proposed by Razafimandimby et al. [22] to efficiently maintain the global connectivity among mobile robots and guarantee the desired Quality of Service level. They define IoRT as a set of devices that can monitor events, fuse sensor data, use local and distributed intelligence to determine the best course of action, and then actuate in the physical world. To carry out cooperative tasks, the robots need to establish ad hoc communication with each other. Thus, maintaining communication among multiple mobile IoRT robots is a crucial issue. To this end, the authors use graph connectivity metric to maintain the global connectivity of IoRT robots’ team, when they are in mobility. The authors claim the proposed algorithms allow the whole IoRT robot network to converge to the desired communication quality.

Psomakelis et al. [18] introduce a platform labelled RADICAL that combines citizens’ posts retrieved through smartphone applications and social networks for smart city services. RADICAL enables to collect, combine, analyse, process, visualise, and provide uniform access to big data sets of social network content, such as tweets, sensor measurements, or citizens’ smartphone reports. In the authors’ view, a social network is a network of recommendations among a circle of “virtual” friends via an online platform.

Lee et al. [29] combine big data and social sensors to create a novel early warning system for dengue outbreaks. Their starting assumption was that environmental sensors were not as ubiquitously deployed as needed for situational awareness. Thus, to deal with the problem, messages of social sensors (i.e., people) and real-time web information (e.g., tweets) were used to detect dengue outbreaks. Their approach was a useful resource for situational awareness, and for investigating ways of understanding how the temporal trend of collected data correlates with the incidence of dengue as identified by national health authorities. Indeed, what the authors label as social messages is considered to contain information which is valuable for understanding the development of real-world events, thus contributing to solutions for event awareness and crisis management. In the study, the information obtained by social sensors (humans) was used for monitoring outbreak events, in combination with the data collected by physical sensors.

Nakashima et al. [21] suggest that a range of information related to the real world can be retrieved through analysis of texts posted on social networks systems (SNS). They consider the acquisition of such information as social sensor usage and refer to the generated data as social sensor data. The authors designed and implemented a system called S-cube to generate and share such social sensor data, along with related analytical programs. Here, tweets are classified into various classes using SVM techniques, and additional analytical programs for generating social sensor data were also considered useful in developing new types of social sensors. Their database stores both texts and images, which can be analyzed by programs.

Finally, Giridhar et al. [31] developed an algorithm to identify and geo-locate real-world events that may be presented as social activity signals on social networks. They focus on content shared by users on both Twitter and Instagram to design a system capable of fusing data across multiple networks. Their fusion algorithm enables the detection of events from various social networks to improve the accuracy of results. Here, Twitter is considered to be a sensor network, and its users are the sensor nodes that report witnessed events according to some probabilistic distribution. Thus, every tweet is a sensor reading, and the probability of event occurrence is expressed with the help of an exponential distribution. The authors solve the problem of corroboration by trying to map description of the events across the two different networks with the support of an unsupervised approach. By combining data from multiple social media, they can detect events that may not have enough corroboration in one network or be indistinguishable “irrelevant news” in another.

Given the focused yet comprehensive review of techniques and approaches presented in this section, in the next section, we identify the main pending challenges.

## 4. Open Challenges and Research Opportunities

**Figure 3:**
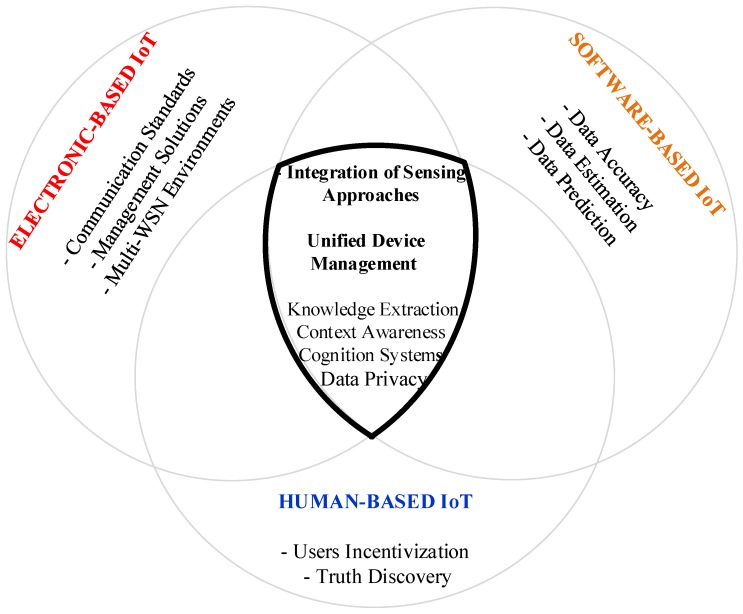
Main open issues per sensing approach.

There are challenges for each sensing approach and challenges that pertain to the confluence of the three sensing paradigms, as depicted in Figure 3 and as will be explained below.

### 4.1. Data Accuracy

Virtual sensing may need to resort to algorithms and techniques for data estimation and/or data prediction. These pose the challenge of data accuracy. In [15], the authors test a temperature virtual sensor framework in a real scenario to estimate values for days or months. With their solution, up to 95% of the sensor readings achieve estimation error within 0.5 °C. According to the authors, their solution achieves a significantly higher accuracy compared to state-of-the-art methods, which are suitable for estimating sensor data for a limited timeframe only, ranging from a few minutes to a few hours. Even though the results are useful for indirect sensing, there is clearly room for improvement. Other virtual sensing solutions, e.g., [34], have similar limitations, and better approaches to estimation and prediction are needed.

The main challenge in social sensing applications is the determination of the correctness of the observations from unknown and potentially untrusted data sources [35]. This point is also shared by the authors in [36] and in [37], to whom a critical challenge in social sensing paradigm is how to ascertain the credibility of claims and the reliability of sources without knowing them a priori. These challenges are labelled as truth discovery or fact-finding. In [36], the authors present an analytical model that considers the mood sensitivity feature to solve the truth discovery problem. On the other hand, the authors in [35] develop a plan claimed to solve the problem known as truth discovery. As future work, they plan to integrate the proposed scheme with another platform they have developed and evaluate its performance through several real-world case studies using twitter data feeds. Another example is the work presented in [38], in which its authors developed a dependency model for social network sources that is shown to improve the quality of fact-finding.

### 4.2. User Incentivization

One of the significant challenges concerning social sensing and, more generally, crowdsensing techniques is how to provide incentives for user participation. The increasing popularity of mobile phones social sensors (MPSS) brings new problems to the online social networks (OSN) solution that can affect the users’ motivation to join a community [19,39]. Among such problems, we have significant battery consumption, mobile network financial costs to the phone users, and substantial traffic load, in particular for applications that require fine-grained continuous sensing. Most of the current MPSSs transmit mobile sensor data to servers through cellular networks [33,40]. Using OSNs as higher-level communication channels must consider a conservative behaviour from the users regarding both battery and Central Processing Unit (CPU) consumption [19], as well as communication costs. The authors in [33] defend rewarding phone users to cover sensing and transmission costs, to encourage them to participate in the social system. Finding effective ways for user incentivization is a critical issue for the success of social sensing, especially when it is MPSS-based.

### 4.3. Data Privacy

Data privacy is another major concern and challenge in a socially interactive Internet. Data privacy is a common issue in computing systems, since they all deal with operating on data that are sensible for at least one application. Considering that privacy remains a huge issue for IoT, Yang et al. [41] surveyed user privacy issues in the literature. They present a classification of IoT attacks, describe mechanisms and architectures for authentication and access control, and analyze both the issues and mechanisms in all the IoT communication layers, as previously presented in Table 1. They concluded that all IoT devices could be vulnerable to certain types of attacks, just like any IT system is vulnerable at some point. One of the approaches nominated to mitigate the risk of attacks is the development of security solutions with lightweight computing needs due to the limitations of the electronic-based IoT devices. The second approach is the development of open, standardised security policies for IoT products, because the best safety solutions for commercial IoT devices are often tackled on silos based on the technologies, protocols, and security mechanisms implemented by each vendor.

He et al. [42] analysed the security challenges in IoT-enabled cyber-physical systems, namely, critical infrastructures and industry 4.0. Among the opportunities for future works, they identified investigations into the security architecture for IoT. Indeed, an appropriate security architecture would lead to an implementation of the security by Design, that is, before any installation of IoT-enabled cyber-physical systems. They also identify Data Driven Cybersecurity, where data is recommended to be protected both in static and communication perspectives. Finally, the authors state that many advanced security solutions for traditional systems can be adapted for IoT-enabled cyber-physical systems by leveraging Evolutionary Computation and other Computational Intelligence techniques. 

Even if the management of crowdsourcing platforms is a centralised one, this does not mean that they are willing to give up on their privacy. To the authors in [32], there may be concerns regarding reliability and privacy-preservation when using crowdsensing platforms to deliver services. The problem could become more acute when the social sensing paradigm becomes more widely used. The studies in [43,44] present solutions for the preservation of user privacy in a crowdsensing environment. The solutions are judged to be useful when compared to related work and were validated using real-world datasets. The authors in both studies achieve the results without losing data accuracy or the means to incentivise the users fairly. Like related studies in the literature, the presented solutions rely on a reward-based system, which often has a monetary cost for the service provider.

### 4.4. Wireless Sensor Networks

Even though the authors in [22] propose a graph connectivity metric to maintain global robot connectivity while mobile, they admit that maintaining communication among multiple mobile IoRT robots is still a crucial issue. To overcome many challenges concerning the inefficient utilization of the radio spectrum in IoT, namely on bandwidth allocation to large numbers of devices, recent studies point to the use of Cognitive Radio frameworks in the future [45]. Last but not least, the use of multiple technologies, either standardised or not, in multi-WSN environments is one of the more critical challenges of current WSN applications and systems, from a management point of view. Architectures and solutions that can integrate both multiple standards and multiple networks, are essential for the wide acceptance and effective deployment of WSNs [46].

### 4.5. Integration of Sensing Approaches and Unified Device Management

The combination of the three sensing approaches addressed in this paper—electronics, virtual, and human—with the social capabilities that may be configured for each of them is one of the more prominent challenges for an effective, socially interactive Internet. Large-scale sensing tasks are more likely to get reliable results if sensed data is collected by many sensing approaches [32]. Thus, combining all available sensing approaches results in richer, more efficient sensing, with enhanced contextualised data from a broader set of possibilities. Indeed, high-end sensors could be used by multiple nodes in parallel, instead of requiring separate installations for specific nodes [30]. The proper combination of sensor inputs, while collecting and processing micro-sensors data, is still far from being common [47]. In this regard, the special issue of a recent publication underlines the synergy among the collected data as still a challenging task in IoT [48].

On the other hand, the platforms presented in [18,29] are a first approach to the integration of the three sensing approaches. However, the proposed integration is quite limited and cannot offer an appropriate response to IoT latency-sensitive applications [49,50], besides being entirely dependent on Internet connectivity. In this regard, levering the Mobile Edge Computing [51] paradigm in the integration of sensing approaches can be a new solution design to provide such an appropriate response. More generally, flexible integration approaches are needed, which, in addition to providing some integration, can adequately address other challenges, such as knowledge extraction, context awareness, and cognitive systems. Lastly, this heterogeneity in sensing and actuating approaches leads to the challenging research opportunity of finding a unified management solution for IoT devices, presented in the broadest sense in Section 2. Indeed, providing a management solution that deals with the IoT device layer from the perspective of the confluence of electronic-based, software-based, and human-based sensors and actuators is still an open issue. Yet, proper management of IoT systems is crucial to their operation [7]. Many proposals addressing IoT management issues can be found in the literature but, to the best of our knowledge, they are focused on well-established physical-electronic IoT devices. While many such solutions are proprietary, some standards-based approaches can also be found, aiming at facilitating overall management of IoT systems.

Table 4 provides references to the key challenges we identified in this section, for the three sensing approaches. While the first three columns from the left present the state of the art, in the column “limitations” we show what is still a gap in the literature and thus the main open challenges. In the last column are listed some relevant works from which we can find the claimed limitations in each reference criteria.

## 5. Conclusions

Sensing is changing not only the Internet but also the way people are interacting with it and with other people. What started as environmental sensing in a single, restricted-scope network has quickly grown into an Internet of Things and, currently, is giving way to clouds of virtual sensors and to the social sensing paradigm, in which people and processes are both data producers and consumers. In this context, the current paper provided a survey of sensing approaches in the literature, with emphasis on the 2015–2017 period.

A social sensor is a status that can be assigned to any entity with sensing capabilities, according to the running application. While the literature presents a more general networked view of leveraging sociability in IoT, in this study, we focused on the specificity of the IoT device level. Hence, going beyond physical sensing, we addressed what we call virtual sensing approaches on one side, and social sensing on the other side, and the respective actuation counterparts. It is also important to note that a crowdsensing approach is not necessarily a social sensing paradigm. In the latter, the far-endentities, i.e., the sensors, only produce data that is sent to be consumed remotely, while in the former, sensors are themselves both data producers and consumers.

The subsequent identification of research challenges clearly points to critical open issues concerning the identified three sensing approaches: electronics, virtual, human-based. The challenges in each of the mentioned areas are stimulating. Nevertheless, seamless integration of the various sensing approaches and a unified management for the three built-in natures of the IoT devices, with the associated measurement results from practical implementation, are probably the most significant challenges, as they are crucial to an operational socially interactive Internet. 

## Figures and Tables

**Table 1:** Simplified view of the IoT layered model. Adapted from [7].

Layers and Sub-Layers		Some Enablers
**Application**		The user interface, services
**Middleware**		Modelling languages, data models
**Network**		Nodes, communication supports
**Device**	Networking Capability	Gateways, Proxies
	Sensing/actuating Capabilities	Smart sensors/actuators

**Table 2:** Taxonomy of sensors and actuators in IoT.

Nature of the Interacting World	Built-in Nature	Assigned Relationship Capabilities
Physical	Electronic-based	Standalone Social
	Human-based	
Virtual		
	Software-based	

**Table 3:** Identification of relevant work, organised according to the proposed taxonomy.

Nature of the Interacting World		Built-in Nature of the Sensing/Actuating Entities					Assigned Relationship Capabilities	
Physical	Virtual	EB *	HB **	Software-based			Stand alone	Social
[3,6,7,8,9,11,12,19,21,22,25,26,27,28,29]	[7,13,14,15,16]	[3,6,7,8,9,11,12,22,25,26,27,28,29,30]	[3,6,17,18,19,21,29,31]	Repository	Analytical Results		[6,7,9,11,12,13,32,33]	[6,18,19,21,22,25,26,27,28,29,31]
				[10,11,12,13]	Estimation	Prediction		
					[13,14,15,17,22]	[13,16]		

* Electronic-based; ** Human-based.

**Table 4:** Main open challenges identified in the literature.

Reference Criteria	Research Directions	Potential Solutions	Limitations	Relevant Work
**Data Accuracy**	Data estimation Data prediction Information truth discovery/fact-finding	Analytical cognitive systems Integration of multiple sensing Approaches	Noisy Environments Management of data sources Users privacy	[15,25,34,35,36,38,39,43,44]
**Users Incentivization**	Mobile phones social sensors	Online social networks Users rewarding system	Traffic load and network financial costs Users privacy A reward-based system which often has a monetary cost for the service provider. Data privacy issues	[19,33,39,40,43,44]
	Crowdsensing platforms	Reward-based systems	Limited deployed use cases Quality of the data collected due to heavy traffic load and high-power consumptions Lack of universal Method for crowdsensing strategies A reward-based system which often has a monetary cost for the service provider.	[39,43,44]
**Data Privacy**	Full stack analysis	Static analysis (stored data) Communication analysis (data circulating between communication systems) Design-driven security	Energy and computational capabilities in IoT devices for running more efficient security algorithms General security policy and standards for IoT products Adaptation of advanced security in traditional systems to IoT systems	[41,42]
**WSN**	Bandwidth allocation Connectivity while in mobility	Cognitive radio Network virtualisation	Limited deployed use cases Inefficient utilization of the spectrum	[11,22,45]
	Network management	Management standardisation	Limited deployed use cases Multi-WSN environments	[46]
